# Modulating the metabolism by trimetazidine enhances myoblast differentiation and promotes myogenesis in cachectic tumor-bearing c26 mice

**DOI:** 10.18632/oncotarget.23044

**Published:** 2017-12-08

**Authors:** Lucia Gatta, Laura Vitiello, Stefania Gorini, Sergio Chiandotto, Paola Costelli, Anna Maria Giammarioli, Walter Malorni, Giuseppe Rosano, Elisabetta Ferraro

**Affiliations:** ^1^ Laboratory of Pathophysiology of Cachexia and Metabolism of Skeletal Muscle, IRCCS San Raffaele Pisana, Rome, Italy; ^2^ Department of Molecular and Clinical Medicine (DMCM), C/o Department of Surgery “Pietro Valdoni”, Sapienza University of Rome, Rome, Italy; ^3^ Department of Clinical and Biological Sciences, University of Turin, Turin, Italy; ^4^ Interuniversity Institute of Myology-IIM, Chieti, Italy; ^5^ Department of Therapeutic Research and Medicine Evaluation, Istituto Superiore di Sanita, Rome, Italy; ^6^ Cardiovascular and Cell Sciences Institute, St George’s University of London, Cranmer Terrace, London, UK

**Keywords:** metabolism, myogenesis, C26 mice, cachexia, trimetazidine

## Abstract

Trimetazidine (TMZ) is a metabolic reprogramming agent able to partially inhibit mitochondrial free fatty acid β-oxidation while enhancing glucose oxidation. Here we have found that the metabolic shift driven by TMZ enhances the myogenic potential of skeletal muscle progenitor cells leading to MyoD, Myogenin, Desmin and the slow isoforms of troponin C and I over-expression. Moreover, similarly to exercise, TMZ stimulates the phosphorylation of the AMP-activated protein kinase (AMPK) and up-regulates the peroxisome proliferator-activated receptor gamma coactivator 1-α (PGC1α), both of which are known to enhance the mitochondrial biogenesis necessary for myoblast differentiation. TMZ also induces autophagy which is required during myoblast differentiation and promotes myoblast alignment which allows cell fusion and myofiber formation. Finally, we found that intraperitoneally administered TMZ (5mg/kg) is able to stimulate myogenesis *in vivo* both in a mice model of cancer cachexia (C26 mice) and upon cardiotoxin damage. Collectively, our work demonstrates that TMZ enhances myoblast differentiation and promotes myogenesis, which might contribute recovering stem cell blunted regenerative capacity and counteracting muscle wasting, thanks to the formation of new myofibers; TMZ is already in use in humans as an anti-anginal drug and its repositioning might impact significantly on aging and regeneration-impaired disorders, including cancer cachexia, as well as have implications in regenerative medicine.

## INTRODUCTION

The adult myogenic response plays a key a role in the maintenance of skeletal muscle homeostasis. Typically, adult myogenesis is triggered by skeletal muscle injuries which lead to induction of new myofiber regeneration mainly sustained by resident myogenic satellite cells (SCs), located underneath the basal lamina of myofibers [1–3]. Upon injury, SCs become activated and differentiate into proliferating myoblasts expressing typical myogenic markers such as Desmin, Myf-5 and MyoD [4, 5]. Further differentiated myoblasts, characterized by the expression of other markers such as neonatal isoform of myosin heavy chain (neoMyHC), myogenin and slow-twitch skeletal muscle troponin T and C, stop proliferating and eventually fuse to pre-existing myotubes or to each other to form new myotubes [6, 7].

Alterations of the myogenic response have been associated with several chronic muscle diseases; in muscular distrophies, for example, continuous cycles of degeneration-regeneration lead to the consumption of the reservoir of SCs and the muscle tissue is progressively replaced by connective tissue [8, 9]. The impairment of adult myogenesis efficacy also contributes to the pathogenesis of sarcopenia in the elderly and, most likely, also to muscle wasting occurring in cancer cachexia [10–17]. Recent data show that the defects in regeneration are not only due to a reduced amount of SCs but also to their impaired functionality and myogenic capacity, which have been associated, among the other causes, with alterations in apoptosis, autophagy and reduced activation of specific signaling pathways [18–23, 24–27]. In particular, defective autophagy in SCs results in the accumulation of toxic intracellular debris mainly composed of altered mitochondria which leads to senescence [25, 26]. It has been proposed that, in order to maintain their energy levels and survive in nutrient poor conditions, SCs rely on autophagy and that during the transition from quiescence to activation, sirtuin 1 (SIRT1) is required for autophagy induction in order to meet bioenergetic demands [28].

Energy management and metabolic reprogramming seem to be crucial for stem cell differentiation. In this regard, favoring the oxidative metabolism has been proposed to enhance myogenic capabilities [29–32]. Studies supporting the key role of metabolism in controlling stem cell fate [33–36] also highlight that mitochondrial metabolism pathways lead to reactive oxygen species (ROS) generation and modulate acetyl-CoA and NAD/NADH or AMP/ATP ratios, all of which are known to regulate SC self-renewal and homeostasis [37, 38]. In line with these observations, it has recently been found that, during the transition from quiescence to proliferation, SCs experience a metabolic switch which decreases NAD^+^ levels and SIRT1 deacetylase activity, thus increasing epigenetic histone acetylation and activation of muscle gene transcription [39]. Notably, it has been shown that slow muscles, characterized by an oxidative metabolism, contain a relatively higher number (up to 6 fold) of SCs compared to fast-glycolytic muscles [40, 41].

Based on these premises underlining the relevance of metabolism reprogramming in controlling stem cell fate, we decided to analyze the influence of the metabolic modulator Trimetazidine (TMZ) –a partial inhibitor or free fatty acid β-oxidation able to enhance glucose oxidation– on myoblast survival and differentiative potential. A group of drugs referred to as metabolic modulators has been extensively studied for their beneficial effects on myocardium due to their ability to optimize metabolism [42–46]. In particular, among them, TMZ shifts the energy metabolism from fatty acid oxidation to glucose oxidation by inhibiting the mitochondrial long-chain 3-ketoacyl coenzyme A thiolase. This change optimizes cell metabolism since glucose is a more efficient substrate for ATP synthesis than fatty acids; in fact, fatty acids have a lower P/O ratio compared to carbohydrates since their β-oxidation generates a larger amount of ATP compared to glucose oxidation but at the expense of a greater oxygen consumption [42]. This accounts for a cytoprotective role of TMZ which, as established by a large number of clinical studies, exerts a clear beneficial effect in patients with ischaemic cardiomyopathy; increased exercise tolerance and a decreased angina have been reported compared to placebo. Therefore, TMZ has been approved for its use in humans for the treatment of chronic stable angina as monotherapy and adjunct therapy [42–46]. Notably, TMZ has recently been found to also act on skeletal muscle being able to protect skeletal myotubes *in vitro* from atrophy due to TNFɑ and to serum starvation by triggering the Akt pathway. Moreover, TMZ has also been shown to increase strength in mouse models of aging [47, 48] and in C26 cachectic mice [49].

## RESULTS

### TMZ enhances differentiation of C2C12 myoblasts

**Figure 1:**
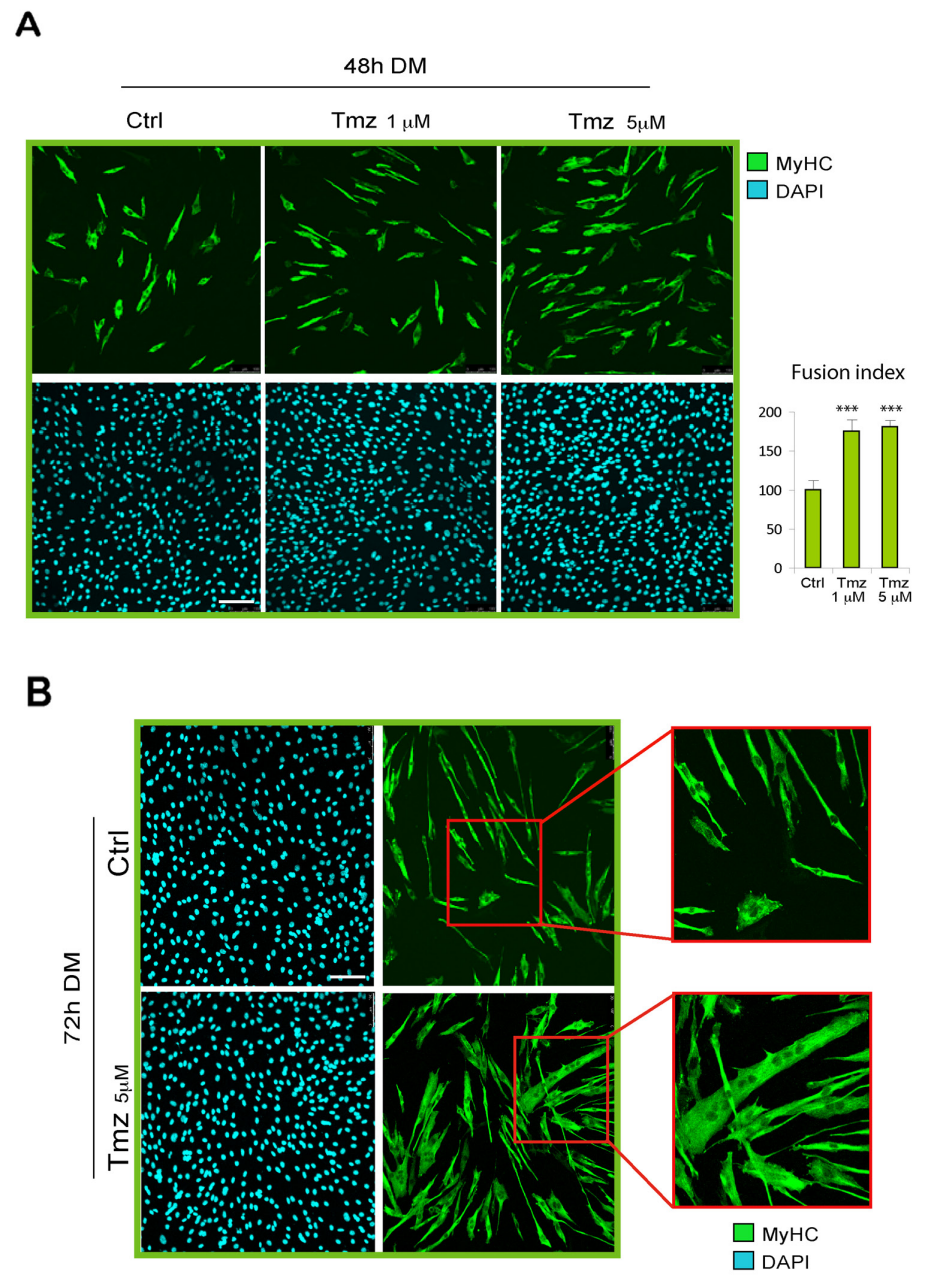
TMZ enhances C2C12 skeletal myoblast differentiation **(A)** Representative pictures of C2C12 myoblasts differentiating for 48 hours in differentiating medium (DM) in the absence (Ctrl) or presence (TMZ) of different concentrations of TMZ and then stained with anti-MyHC antibody (green) and DAPI (blue) for nuclei detection. Quantification of fusion index after these treatments is shown in the histogram on the right. At least 30 fields were analyzed for each condition in four independentexperiments. Data are mean ± s.e.m. ^***^p ≤ 0.005 by two-tailed Student’s *t*-test. Scale bar: 150 μm. **(B)** Representative pictures of C2C12 myoblasts differentiating for 72 hours in DM in the absence (Ctrl) or presence (TMZ) of TMZ. The higher level of fusion with TMZ is highlighted by the enlargement of the red labeled area. Scale bar: 100 μm.

In order to explore the effect of metabolic modulation on skeletal myoblast differentiation, we induced C2C12 myoblasts to differentiate by culturing them on DM in presence or absence of various concentrations of TMZ (1-5-30 μM). We evaluated the extent of differentation after 48h of DM incubation by immunostaining for MyHC used as marker for terminal myogenic differentiation. As can be observed in Figure 1A, the administration of TMZ caused an increase of MyHC-positivity compared to untreated cells. Since the effect of TMZ on C2C12 cells was more reproducible by using 5 μM TMZ we continued using this concentration throughout the present study, unless otherwise stated.

The effect of TMZ on C2C12 differentiation was also evaluated by calculating the fusion index which indicates the formation of multinucleated myotubes. We found that TMZ determines an increase of the fusion index by around 75% compared to untreated cells (Figure 1A, right panel graph). The fusion index was obtained by calculating the average of nuclei in MyHC-positive myotubes (containing more than 3 nuclei) normalized to the total number of nuclei [50]. The increased fusion triggered by TMZ is also evident at 72h of DM incubation (Figure 1B) and longer incubation times display very large myotubes in which the increased fusion effect and the hypertrophic effect of TMZ, previously revealed by treating mature myotubes [47], might have been additive (Supplementary Figure 1).

### Overexpression of myogenic genes is triggered by TMZ

**Figure 2:**
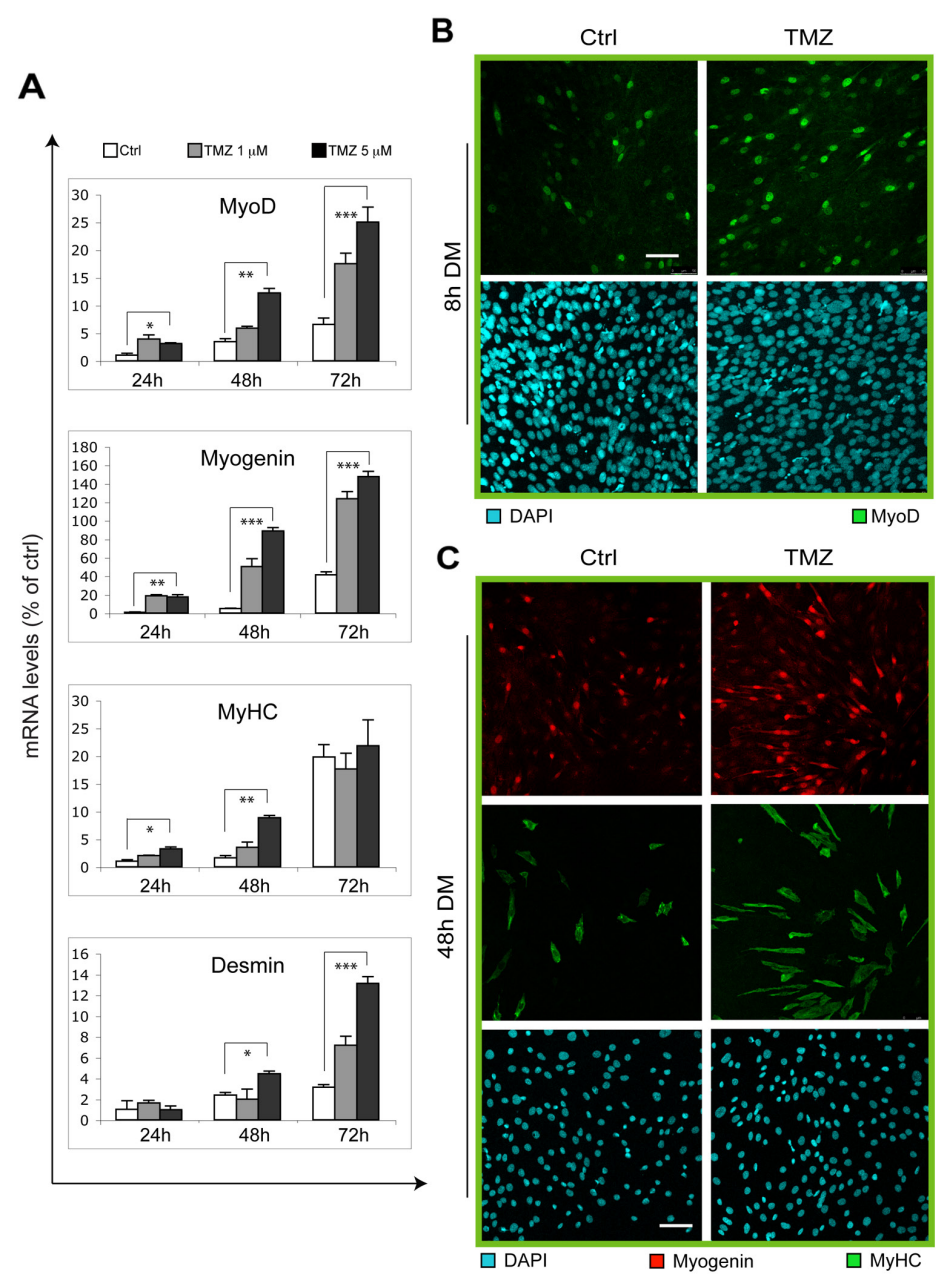
Myogenic genes are overexpressed upon TMZ treatment **(A)** The mRNA levels of MyoD, Myogenin, Desmin and MyHC, were evaluated by quantitative real time PCR in C2C12 differentiating (for 24, 48 and 72 hours in DM) myoblasts treated (TMZ) or not (Ctrl) with 1 μM or 5 μM TMZ. Data were normalized to 18S used as internal control. Data display the percentage of mRNAs relative to untreated cells (24h in DM), which was arbitrarily set as 1. Data shown are the mean ± s.e.m. from three experiments each performed in triplicate. ^*^p ≤ 0.05, ^**^p ≤ 0.01 and ^***^p≤ 0.005 by two-tailed Student’s *t*-test. **(B)** Representative pictures of C2C12 myoblasts differentiating for 8 hours in DM in the absence (Ctrl) or presence of 5μM TMZ (TMZ) and then stained to detect MyoD (green) and nuclei (DAPI, blue). Five experiments were performed. Scale bar: 75 μm. **(C)** Representative pictures of C2C12 myoblasts differentiating for 48 hours in DM in the absence (Ctrl) or presence of 5μM TMZ and then stained to detect Myogenin (red), MyHC (green) and nuclei (DAPI, blue). Five experiments were performed. Scale bar: 75 μm.

To confirm our data suggesting a potentiation of the myogenic program by TMZ, we analyzed the expression levels of some muscle-specific genes during differentiation with or without supplementation of TMZ (Figure 2A). In accordance with our observations reported in Figure 1, we found that TMZ triggers a marked increase of MyoD, Myogenin and Desmin transcript levels at 24h, 48h and 72h from the onset of differentitation. MyHC also increases after 24h and 48h of DM-TMZ incubation. The effect of TMZ was also evaluated at protein level by performing the immunofluorescent detection of MyoD and Myogenin after 8h and 48h, respectively, from DM incubation. As shown in Figure 2, the number of nuclei positive for MyoD or Myogenin increases upon TMZ administration (Figure 2B, 2C). These results clearly indicate a TMZ-dependent enhancement of the differentiation programm in C2C12 cells.

### Exposure to TMZ promotes a parallel orientation of myotubes

**Figure 3:**
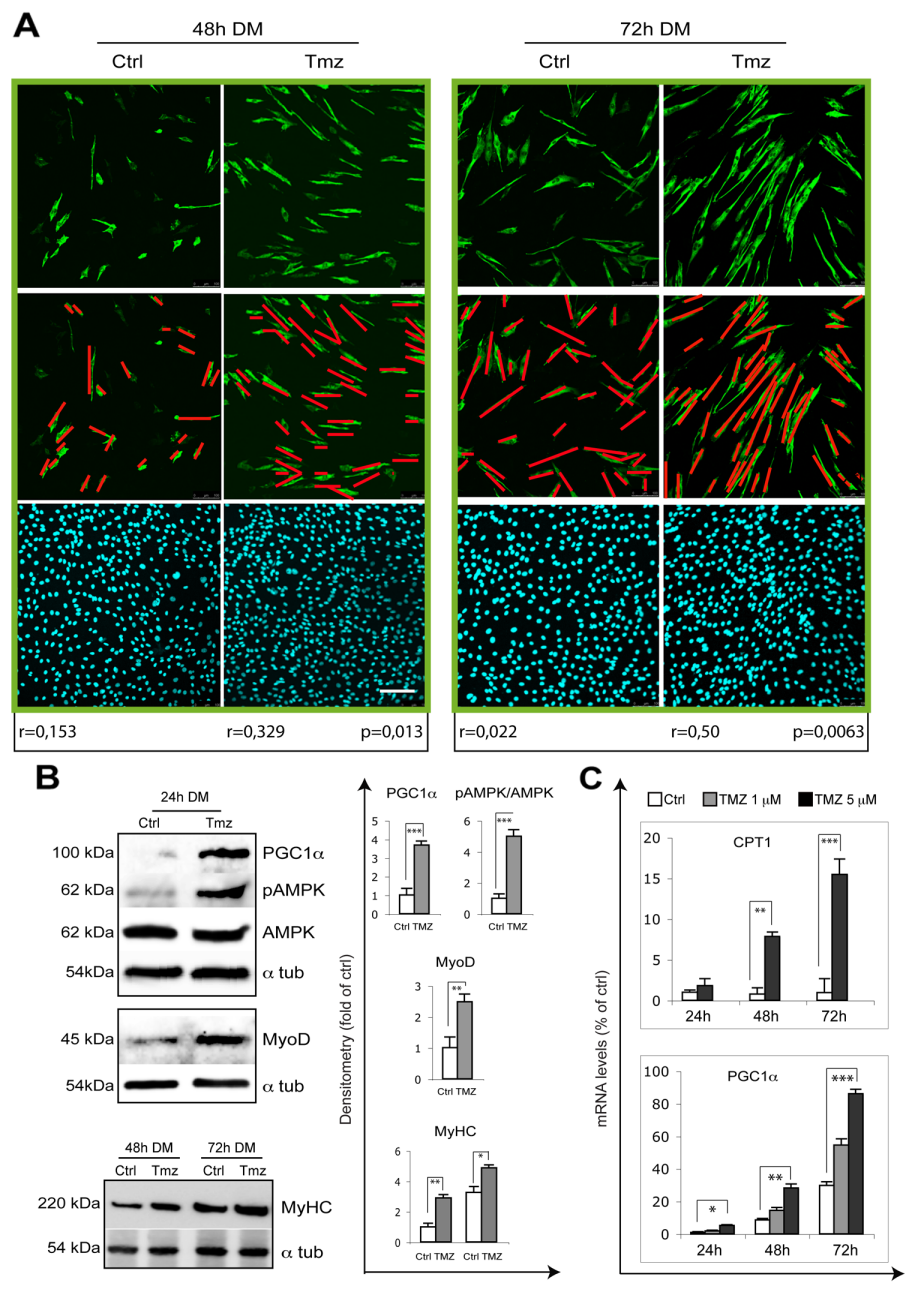
TMZ enhances the alignment of differentiating myoblasts **(A)** Representative pictures of C2C12 myoblasts differentiating for 48 hours and 72 hours in DM in the absence (Ctrl) or presence of 5 μM TMZ (TMZ) and then stained to detect MyHC (green) and nuclei (DAPI, blue). Scale bar: 150 μm. n = number of analyzed cells; r = mean vector length (statistic of Rayleigh test); p = observed significance. The mean vector length r can range from 0 (perfect isotropy; of multiple cell directions) to 1 (perfect anisotropy; existence of a preferred-aligned-cell direction). Three independent experiments for each condition were performed, with a total of not less than 300 cells analyzed for each condition. **(B)** Extracts from untreated control C2C12 (Ctrl) and 10μM TMZ-treated C2C12 cells (TMZ) were assayed for MyoD, pAMPK, AMPK, PGC-1α and MyHC protein levels. Protein levels of a representative experiment out of three are shown. α-tubulin was used as loading control. Density of immunoreactive bands was calculated using the ImageQuant TL software from GE Healthcare Life and normalized for α-tubulin. Each value indicates the mean± s.e.m. (reported as percentage of Ctrl) of the densitometric analysis on three independent immunoblots. ^*^p ≤ 0.05, ^**^p ≤ 0.01 and ^***^p≤ 0.005 by student *t*-test. **(C)** The mRNA levels of PGC-1α and CPT1, were evaluated by quantitative real time PCR in C2C12 differentiating (for 24, 48 and 72 hours in DM) myoblasts treated (TMZ) or not (Ctrl) with 1 μM or 5 μM TMZ. Data were normalized to 18S used as internal control. Data display the percentage of mRNAs relative to untreated cells (24h in DM), which was arbitrarily set as 1. Data shown are the mean ± s.e.m. from three experiments each performed in triplicate. ^*^p ≤ 0.05, ^**^p ≤ 0.01 and ^***^p≤ 0.005 by two-tailed Student’s *t*-test.

The correct orientation of myocytes is crucial for myotube formation and a correct myofiber orientation allows a longitudinal contraction of the muscle. Cell alignment, which is the parallel apposition of the long axes of myoblasts/myocytes, precedes fusion. We observed that TMZ affects myocyte orientation by enhancing the parallelism among cells, this possibly improving fusion and promoting myotube formation (Figure 3A). Moreover, similarly to a previous report showing that the application of a static magnetic field to C2C12 cells enhances the parallelism among cells and also induces myotube hypertrophy [51], we observed an increase of the diameter of myotubes treated with TMZ (Figure 1B and 3A). We therefore propose that exposure to TMZ also stimulates myotube differentiation by affecting myocyte orientation. MyoD and MyHC protein levels also increase in the presence of TMZ (Figure 3B). TMZ being a metabolic modulator, we evaluated the activation of the energetic sensor AMPK which, interestingly, resulted over-phosphorylated (Figure 3B). In line with our recent observations in mice [49], we also found an up-regulation of the peroxisome proliferator-activated receptor gamma coactivator 1-alpha (PGC1ɑ) transcript levels in the presence of TMZ and an increase of the mitochondrial transporter of fatty acids, carnitine palmitoyl transferase 1 (CPT1) transcript (Figure 3C), possibly as a compensative response to the reduction of free fatty acid β-oxidation. Accordingly, also PGC1ɑ protein was over-expressed by TMZ treatment (Figure 3B).

### TMZ does not affect C2C12 proliferation rate, apoptosis and ROS levels

**Figure 4:**
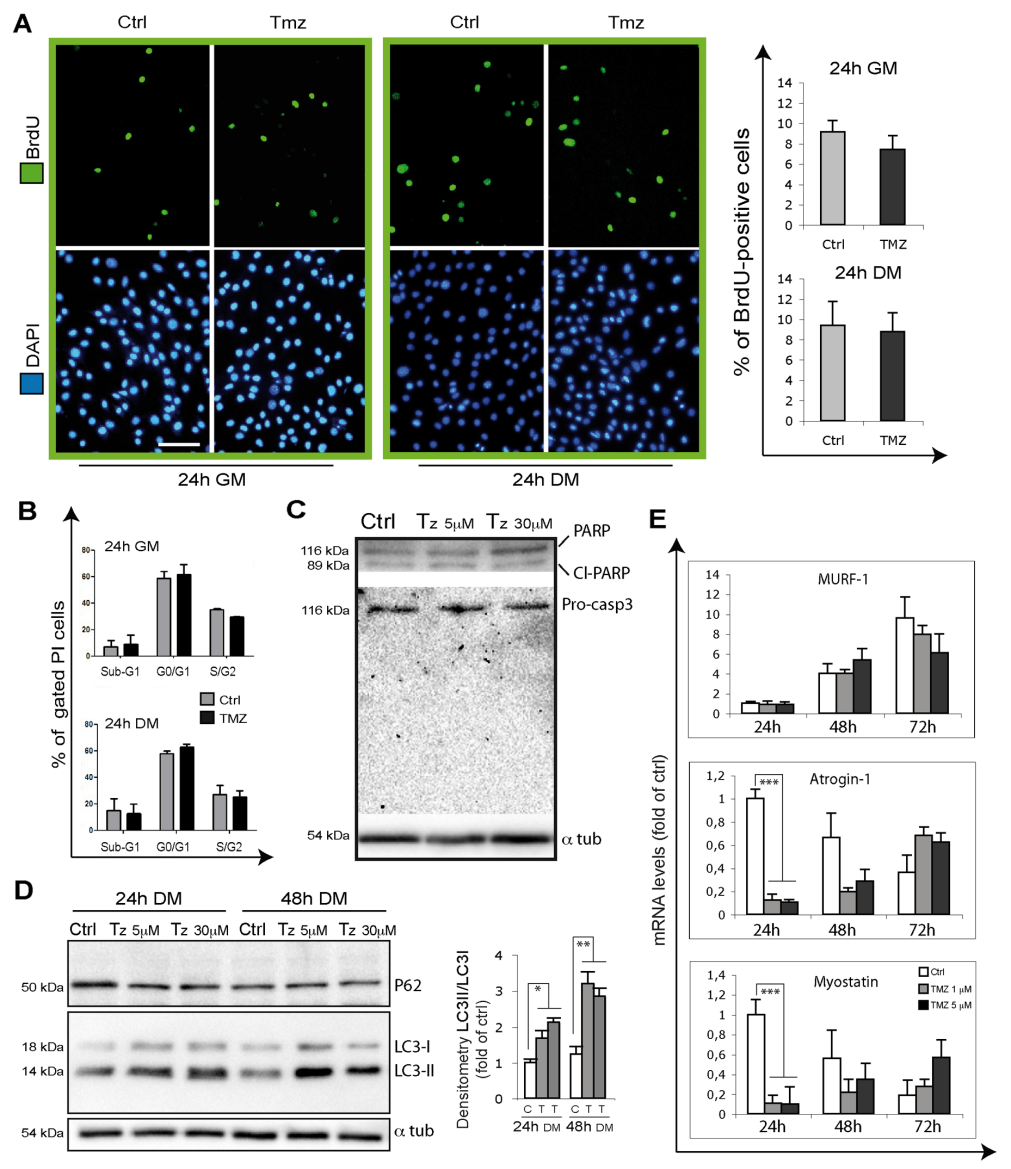
TMZ influence on proliferation, apoptosis, autophagy and atrophy markers **(A)** Representative images of BrdU incorporating C2C12 cells (green) treated for 24 hours with or without 5 μM TMZ in GM and in DM. During the last 4h of incubation, cells were labeled with BrdU. Nuclei were stained with DAPI (blue). Four experiments were performed. Scale bar: 75 μm. **(B)** Cell cycle analysis of C2C12 in GM or DM for 24 hours with or without 5 μM TMZ. Cells were then stained with propidium iodide. Histograms show the mean ± SE of four independent experiments. No difference were observed in resting (G0/G1) and proliferating cells (S/G2) nor in the percentage of hypodiploid apoptotic (sub-G1) cells. **(C)** Total extracts of differentiating myoblasts kept for 24 hours in DM untreated (Ctrl) or TMZ-treated (5 μM and 30 μM) were assayed for PARP and Caspase-3 cleavage and **(D)**, also kept for 48 hours in DM, for LC3 and p62. The cleaved form of Caspase-3 is undetectable in each condition. Protein levels of a representative experiment out of three are shown. ɑ-tubulin was assayed as a control for equal protein loading. **(E)** The mRNA levels of Atrogin-1, Murf1 and Myostatin were evaluated by quantitative real time PCR in differentiating (for 24, 48 and 72 hours in DM) C2C12 treated or not (Ctrl) with 1 μM or 5 μM TMZ. Data were normalized to 18S. Data display the percentage of mRNAs relative to untreated cells for 24 hours in DM, which was arbitrarily set as 1. Data shown are the mean ± s.e.m. from three experiments each performed in triplicate. ^***^p≤ 0.005 by two-tailed Student’s *t*-test.

In order to evaluate the extent of cell proliferation whose alteration might also affect the number of differentiating cells, we evaluated 5-bromo-2’-deoxyuridine (BrdU) incorporation on C2C12 myoblasts treated or not with TMZ for 24h, both in GM and in DM. In neither case did we observe a different rate of proliferation (as shown by the % of BrdU-positive cells) in TMZ-treated cells compared to their control counterparts (Figure 4A).

To confirm these data we also performed cell cycle analysis by propidium iodide (PI) staining and FACS analysis in GM- and DM-incubated C2C12 cells in presence or absence of TMZ for 24h. No differences were observed in the percentage of resting cells (G0/G1) nor in that of proliferating cells (S/G2), this confirming that TMZ does not affect the rate of cell proliferation (Figure 4B). Of note, we obtained the same results by treating the cells with TMZ for 48h (Supplementary Figure 3).

**Figure 5:**
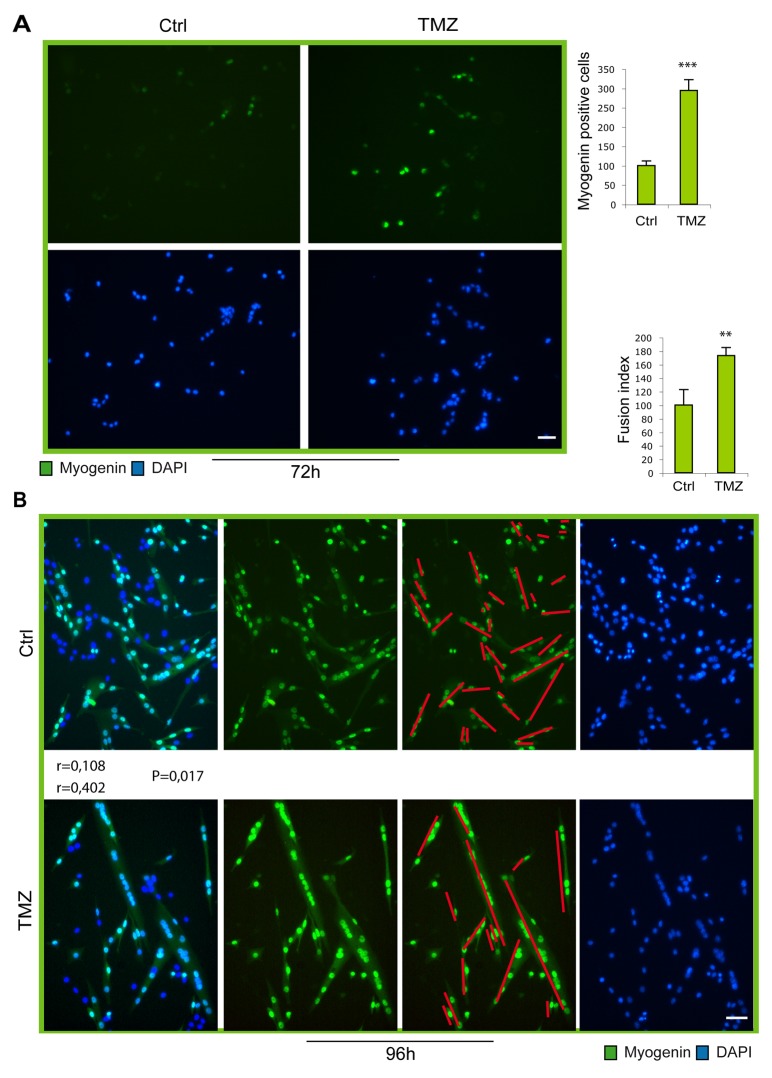
Myogenic gene expression and fusion increase in TMZ-treated SCs **(A)** Representative images of satellite cells in culture for 72 hours, the last 48 hours of which treated (TMZ) or not (Ctrl) with 50 μM TMZ for the last 48 hours. Our previous esperiments showed that a higher concentration of TMZ is needed for the treatment of SCs compared to C2C12 cells. Cells were incubated with an anti-myogenin antibody (green) and with DAPI (blue). Scale bar: 75 μm. **(B)** Representative images of satellite cells in culture for 96h the last 48h of which treated (TMZ) or not (Ctrl) with 50 μM TMZ for the last 48 hours. Cells were incubated with an anti-myogenin antibody (green) and with DAPI (blue). Quantification of fusion index is shown in the histograms on the right. At least 20 fields were analyzed for each condition in four independentexperiments. Scale bar: 75 μm. Data are means ± SEM. ^**^P < 0.01 and ^***^P < 0.005. For myotube orientation measurement, three independent experiments for each condition were performed, with a total of not less than 300 cells analyzed for each condition. n = number of analyzed cells; r = mean vector length (statistic of Rayleigh test); p = observed significance. The mean vector length r can range from 0 (perfect isotropy; of multiple cell directions) to 1 (perfect anisotropy; existence of a preferred-aligned-cell direction).

These experiments also revealed that the sub-G1 cell population, which correlates with the percentage of dead cells, does not significantly vary with TMZ (Figure 4B). This is also evident in Figure 1, 3, 4 and 5A, where the analysis of the morphology of DAPI-stained nuclei shows the absence of picnosis and fragmented nuclei as signs of apoptosis, due to TMZ. In order to further assess the extent of apoptosis, we also evaluated active Caspase-3 and PARP cleavage by WB analysis and, as expected, we found no difference between TMZ-treated (both at 5 μM but also at the higher concentration 30 μM) and untreated C2C12 myoblasts (Figure 4C). The cleaved form of Caspase-3 is not detectable anywhere. Finally, we measured ROS production, which might affect cell viability, by DCF staining and flow cytometry in C2C12 myoblasts. This analysis showed that TMZ incubation for 1h and for 24h does not alter ROS levels (Supplementary Figure 3). We can therefore conclude that TMZ neither alters cell proliferation rate nor does it induce apoptosis or ROS production in C2C12 cells during differentiation.

### Atrophy genes and autophagy are influenced by TMZ in differentiating C2C12

Based on the recently suggested relevance of autophagy to myoblast differentiation [52], and given that TMZ is able to induce autophagy in mature myotubes [47], we also analyzed autophagy during myoblast differentiation by determining LC3-II protein levels and the LC3-II/LC3-I ratio which we found to be increased in the presence of TMZ during C2C12 differentiation (Figure 4D). Additionally, a deeper analysis of the effects of TMZ during C2C12 differentition shows that the transcript levels of factors involved in myotube hypotrophy, namely atrogin-1/MAFbx and myostatin, decrease in TMZ-treated C2C12 myoblasts cultured in DM for 24h, to progressively return to control levels at 48h and 72h (Figure 4E), while MuRF-1 mRNA levels are not affected by TMZ in the same conditions (Figure 4E). We speculate that, since TMZ inhibits NF-kB [53, 54] which is related to Atrogin expression, this might cause a reduction of Atrogin [55]. However, this point needs to be elucidated by additional studies. These data confirm that our findings showing atrophy genes down-regulation and autophagy stimulation evoked by TMZ in C2C12 myotubes [47] also occur during myogenic differentiation.

### TMZ accelerates myogenic maturation of SCs

**Figure 6:**
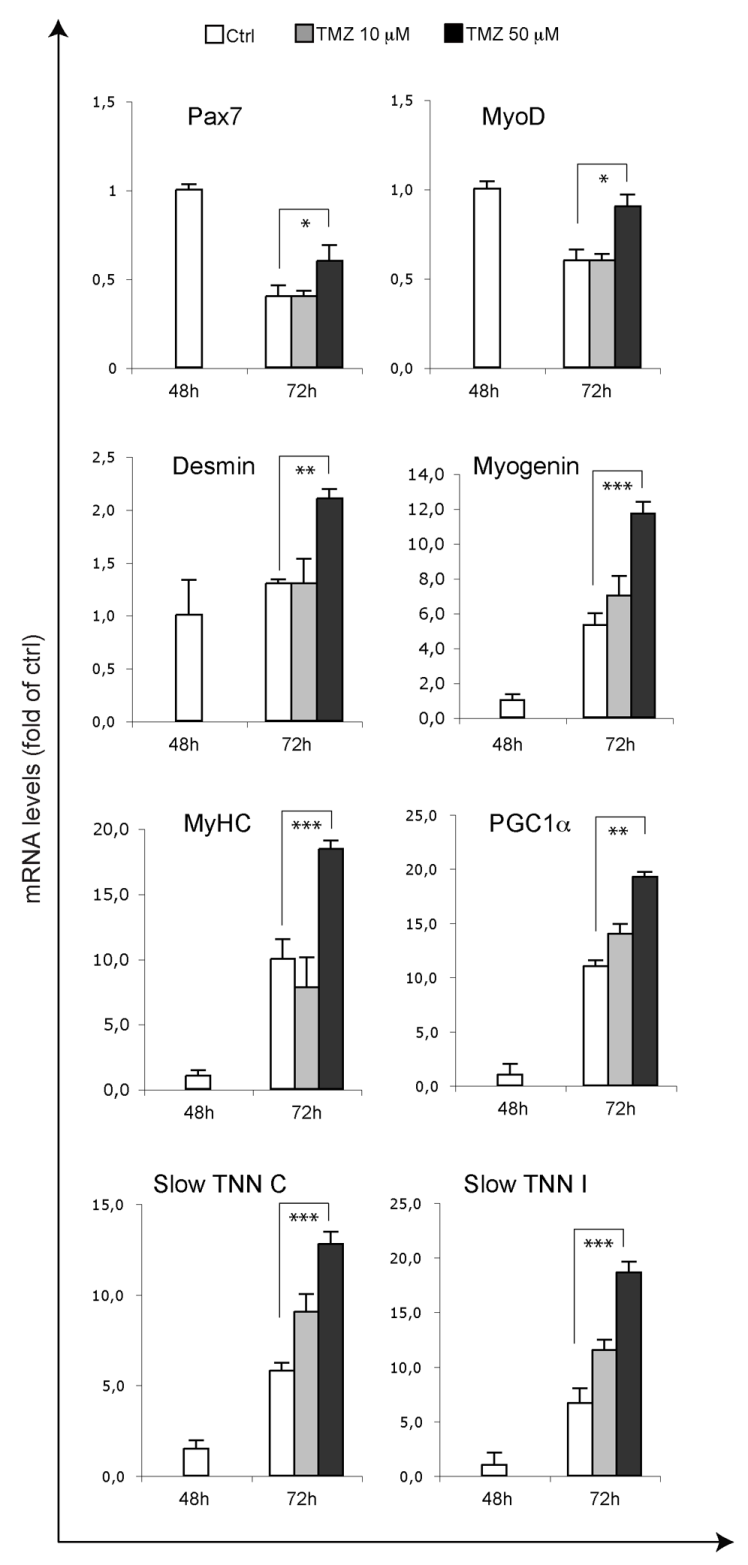
Myogenic genes are overexpressed upon TMZ treatment in SCs The mRNA levels of MyoD, Pax7, MyoD, Desmin, Myogenin, MyHC, PGC1ɑ, slow Troponin C (TNN C) and slow Troponin I (TNN I) were evaluated by quantitative real time PCR in primary SCs differentiating for 48 hours and 72 hours and, in the case of SCs differentiating for 72 hours, treated for the last 24 hours with 10 μM or 50 μM TMZ. Data were normalised to 18S used as internal control. Data display the percentage of mRNAs relative to control 24 hours DM, which was arbitrarily set as 1. Data shown are the mean ± s.e.m. from four experiments each performed in triplicate. ^*^p < 0.05, ^**^p ≤ 0.01 and ^***^p≤ 0.005 by two-tailed Student’s *t*-test.

In order to investigate whether the ability of the metabolic modulator TMZ to accelerate myogenic differentiation is extended also to primary cells, we treated freshly isolated murine SCs with TMZ. Purity of SCs ranged from 88% to 99% (Supplementary Figure 2). As can be observed by immunofluorence staining, at early time points of differentiation (Figure 5A; 72h), the number of SCs expressing myogenin increases upon TMZ treatment, compared to untreated cells. In accordance with our observations obtained in C2C12 cells, at later time of differentiation (96h), the level of fusion in TM-treated cells is markedly higher (73%) than in control cultures (Figure 5B). Notably, we also observed that TMZ-treated SCs were more aligned. Finally, Desmin, Myogenin and MyHC transcript levels significantly increased after TMZ treatment while the increase was moderate for Pax7 and MyoD (Figure 6). Interestingly, also PGC1ɑ and the slow isoforms of Troponin C and I (TNN C and TNN I), which are known to be over-expressed during differentiation, are up-regulated by TMZ; this further indicates a boosting of the differentiative potential triggered by TMZ.

### TMZ stimulates *in vivo* myogenesis both upon cardiotoxin (CTX) damage and in cachectic mice

**Figure 7:**
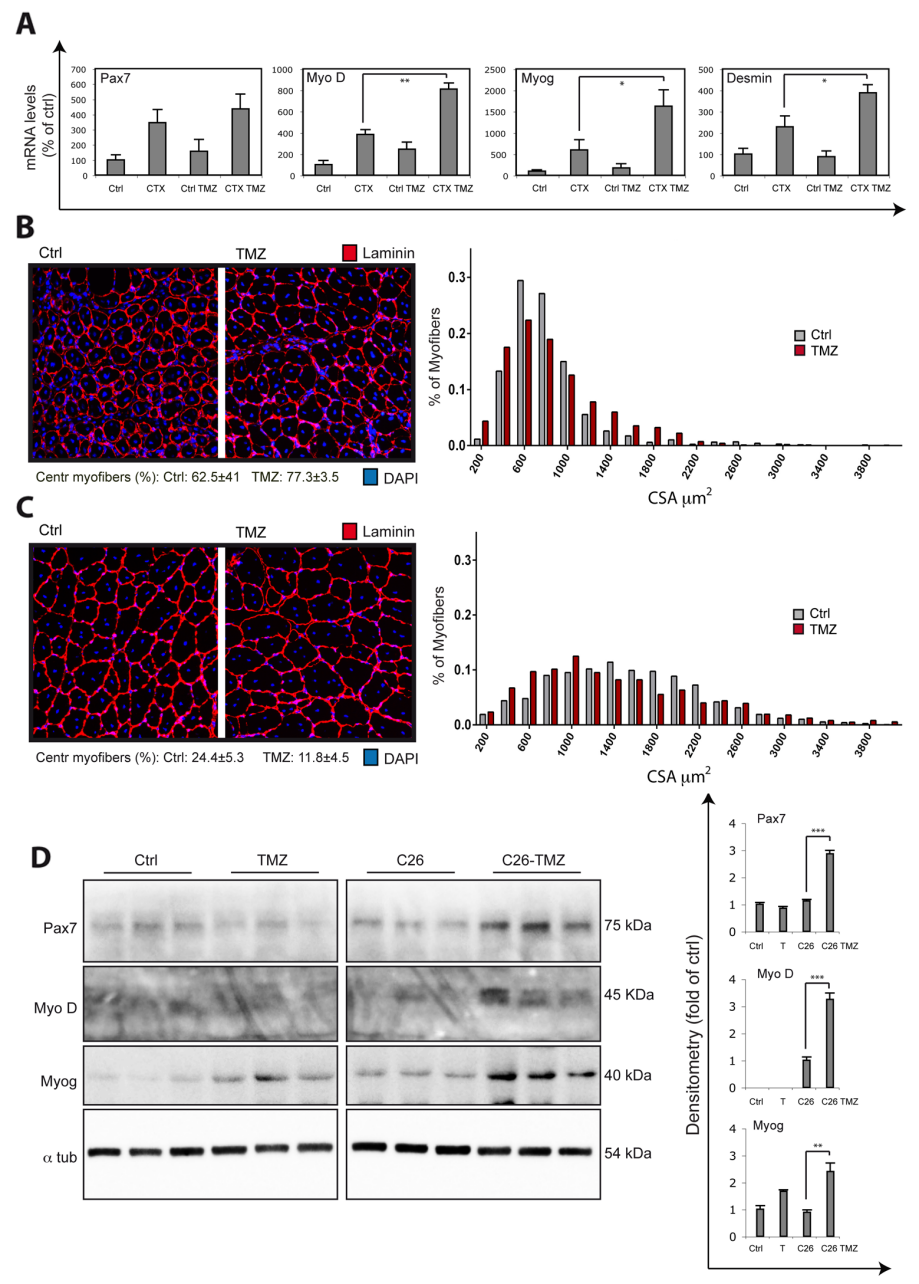
TMZ stimulates new myofiber formation *in vivo* **(A)** The mRNA levels of Pax7, MyoD, Myogenin (Myog), Desmin and MCP1 were evaluated by quantitative real time PCR and were normalised to 18S used as internal control in TA muscles of untreated mice (Ctrl), cardiotoxin (CTX)-injured mice (CTX), TMZ-treated mice (Ctrl TMZ), and TMZ-treated CTX-injured mice (CTX TMZ) 5 days post-injury. At least six mice for each group were used. TMZ was administered 5mg/kg for 5 days by daily intraperitoneal (i.p.) injections from -1 to +4 days with respect to CTX muscle injury date T0. Data display the percentage of mRNAs relative to control, which was arbitrarily set as 100. Data shown are the mean ± s.e.m. from three experiments each performed in triplicate. ^*^p ≤ 0.05 and ^**^p ≤ 0.01 by two-tailed Student’s *t*-test. **(B)** To quantify regeneration due to CTX-induced injury, muscle sections of the injected muscles were analyzed 5 days post-injury. Representative images only of the area with centronucleated myofibers of Laminin/DAPI stained cross-sections from at least three separate transverse planes of TA muscles damaged with CTX untreated (Ctrl) or treated (TMZ) with TMZ are shown. The percentage of centronucleated myofibers (Centr myofibers) on the whole section is indicated. Five untreated mice and and five TMZ-treated mice and at least 5000 myofibers from 5 untreated mice and at least 5000 myofibers from 5 TMZ-treated mice have been evaluated for the determination of cross-sectional area (CSA). Frequency histograms show the distribution of CSA measured only for centronucleated myofibers. Scale bar: 100 μm. **(D)** Same as **(C)** but at 15 days post-injury. The percentage of centronucleated myofibers (Centr myofibers) on the whole section is indicated (^**^p ≤ 0.01). (D) GSN extracts from untreated control mice (Ctrl), TMZ-treated control mice (TMZ), C26 mice (C26) and TMZ-treated C26 mice (C26-TMZ) were assayed for Pax7, MyoD and Myogenin (Myog) protein levels. Protein levels of representative 3 out of 6 mice are shown. A lane between lane 6 and lane 7 was removed from the blot because of technical problems. ɑ-tubulin was used as loading control. Density of immunoreactive bands was calculated using the ImageQuant TL software from GE Healthcare Life normalized for α-tubulin. Each value indicates the mean± s.e.m. (reported as percentage of Ctrl) of the densitometric analysis on three independent immunoblots. ^**^p ≤ 0.01 and ^***^p ≤ 0.001 by Student *t*-test.

Given the effect of TMZ on myoblast differentiation *in vitro*, we evaluated if TMZ was able to improve regeneration upon damage; for this purpose, muscle injury was induced in C57BL6 mice by CTX injection [56] and the animals were exposed to TMZ. At day 5 post-CTX injection, we found that Myo D, Myogenin and Desmin mRNA levels were significantly higher in TMZ treated CTX mice (CTX TMZ) compared to untreated CTX mice (CTX), suggesting that TMZ promotes myogenesis (Figure 7A). The levels of Pax7 do not change significantly and, notably, the levels of the pro-inflammatory cytokine MCP1 (Monocyte Chemoattractant Protein-1) tend to decrease following TMZ treatment, this excluding that the higher myogenesis we found upon TMZ-treatment is caused by a deleterious effect of this drug (Supplementary Figure 4).

We then evaluated the cross-sectional area (CSA) of regenerating myofibers identified by the presence of central nuclei. We found that, at day 5 after CTX injection, the frequency histograms of the centronucleated fiber area delineate a different size distribution in muscles of TMZ-treated *vs* untreated mice; in detail, in TMZ-treated animals there is a higher number of very small centronucleated myofibers (Figure 7B; see fibers smaller than 400 μm, on the left side of the histogram), but also a higher number of very large centronucleated myofibers compared to untreated mice (Figure 7B; see fibers bigger than 1000 μm, on the right side of the histogram). To explain this behaviour, we hypothesize a double action of TMZ which, on one side, might be able to accelerate the growth in size of regenerating myofibers (e.g. by promoting a higher fusion rate and this might be why we observe more big fibers compared to untreated mice), and, on the other side, might keep promoting new myofibers formation (very small fibers occurring). This second potentiating effect on new myogenesis correlates with the enhanced expression of myogenic transcripts (Figure 7A). This bimodal distribution of myofibers also occurs later on, at day 15 post-CTX injection (Figure 7C), although, at this later stage, the percentage of centronucleated myofibers significantly decreases with TMZ compared to Ctrl (Figure 7C) and the frequency histogram on the total number of myofibers shows a shift toward the right side of the graph with TMZ (Supplementary Figure 5A), which mainly supports the acceleration effect of TMZ on muscle regeneration. Moreover, the levels of MyoD, myogenin and eMHC mRNAs are also higher in TMZ-treated than in control mice at day 10 post-injury (data not shown) when, accordingly, the frequency histogram, both on centronucleated and on the total number of myofibers, shows a shift toward the left side of the graph (Supplementary Figure 5B and data not shown), supporting the hypothesis that TMZ also triggers a stronger myogenesis, mainly at earlier time points. A relevant hypertrophic effect of TMZ on myofibers of not damaged muscles has been excluded by our previous work [49] and has now been confirmed by CSA analysis of controlateral muscles at day 15 post-CTX injection (data not shown).

An impaired regenerative response has been evoked as contributing to muscle mass loss in cachexia [14, 15, 17]. In order to clarify the effect of TMZ in pathologies characterized by an altered regenerative machinery, we evaluated the activation of myogenesis in an *in vivo* model of cancer cachexia (C26-bearing mice). Interestingly, we observed an up-regulation of MyoD and Myogenin in TMZ-treated C26 hosts (Figure 7D), suggesting that TMZ is able to release, partially, at least, the impaired myogenesis occurring in C26-bearing mice also without decreasing Pax7 [14, 15], and to promote new myofiber formation, this being potentially protective against muscle wasting. Indeed, Pax7 is not only required to promote the proliferation of undifferentiated cells, but is also necessary for the myogenic differentiation to proceed [14, 57].

Our experiments indicate that TMZ enhances the skeletal muscle regenerative process, both by accelerating it and by keeping stimulating new myofiber formation, while decreasing inflammation.

## DISCUSSION

The present study shows that TMZ exerts a profound effect on myogenic precursor cells by altering their gene expression profile and supporting and accelerating their differentiation *in vitro*, while enhancing myogenesis *in vivo*. These effects likely depend on the metabolic shift and metabolism optimization induced by the drug [42]. This is consistent with the knowledge that the differentiation of myoblasts is associated with a metabolic shift from a predominantly glycolytic state to a metabolism based mainly on mitochondrial oxidative phosphorylation; this shift is thought to be needed to support the higher energetic demand of contractile muscle [52].

Modulation of metabolic pathways is known to regulate SC homeostasis and fate; SC niche environment modification influences SC metabolism, this inducing, along with the triggering of other signaling pathways, SC activation. We found that the metabolic changes induced by TMZ have a similar effect on SCs by potentiating their myogenic program. Similarly, the reprogramming of SC metabolism due to nutrient-dependent changes driven by caloric restriction (CR) or by exercise, has been found to increase SC myogenic activity, both in young and old mice, in concert with an increase in mitochondrial biogenesis and metabolic regulators [31, 32, 58]. In fact, the effects of both CR and exercise appear to be mediated by the energy sensor AMPK that acts on mitochondrial regulators such as SIRT1 and PGC1ɑ [32, 37]. PGC1ɑ is a key regulator of the exercise-mediated muscle adaptation and our experiments showing that TMZ is able to promote PGC1ɑ activation, as well as AMPK over-phosphorylation, (which is known to positively regulate PGC1ɑ) also support our recent observation that TMZ produces, *in vivo*, effects similar to those induced by exercise [49]. Moreover, being SCs activated by exercise, our findings revealing a TMZ-induced enhancement of SC myogenic activity *in vivo* with production of new myofibers, further confirm the exercise-like effect of TMZ and thus its potential beneficial effects [59–62]. However, differently from our results, spontaneous physical activity in C26-bearing mice has recently been shown to reduce Pax7 expression, although no increased myogenesis (which is triggered by TMZ) was reported in this study [16]. In this context, it is relevant to underline that the effect of exercise might vary depending on its type and intensity. In fact, muscle micro-injuries that follow exercise induce a positive compensatory adaptation by an increase in SC activation [58, 63, 64]. However, if the intensity of exercise and damage is too strong, this might be deleterious for the muscle. Exercise mode and intensity need to be optimized in order to achieve the optimal modulation of SC activation, proliferation and differentiatiation [64]. Similarly, we also hypothesise that the concentration and timing of TMZ treatment must be moderate in order to be beneficial; in such a case, the activation of myogenesis by TMZ might contribute protecting skeletal muscle against the loss of mass in different pathologies, including cancer cachexia, and in ageing thanks to the formation of new myofibers. Moreover, in some conditions, a physiologically low response of muscle to exercise occurs, therefore, drugs mimicking some exercise-induced effects might potentially be more adequate in such cases.

Recent studies show that during myoblast differentiation the mitochondrial network gets remodeled by a first stage of mitochondrial fragmentation and clearance by autophagy followed by mitochondrial biogenesis mediated by PGC1ɑ [52]. Moreover, PGC1ɑ is normally up-regulated during myoblast differentiation and its overexpression is able to promote early muscle fiber formation *in vitro* [65]. The ability of TMZ to trigger PGC1ɑ would help sustain mitochondrial biogenesis necessary during differentiation which would enhance myofiber formation capacity.

Moreover, during differentiation of myoblasts into mature myotubes autophagy is robustly upregulated [52]. Our data show that the enhancement of autophagy is also induced by TMZ which is another process by which this metabolic modulator might contribute to accelerate myogenesis. Moreover, in some regeneration-impaired conditions, SC decline in number and functionality has been associated to alterations in autophagy [25, 26, 28]. Indeed, it has been found that promoting autophagy in geriatric mice reverses stem cell senescence and restores regeneration. Here we propose that the boosting effect on SC autophagy, PGC1ɑ expression and differentiative potential induced by TMZ might be potentially useful to improve the muscle myogenic capacity with production of new myofibers.

The enhancement of the myogenic potential and the reduction of the inflammatory citokine MCP1 during regeneration achieved by TMZ treatment, also make this drug an interesting candidate for enhancing the efficiency of skeletal muscle tissue engineering. Consistently, we have also found that exposure to TMZ affects myoblast orientation and promotes alignment [66]. Notably, tools able to control cell orientation are extremely relevant for correct artificial skeletal muscle engineering [67]. TMZ seems to enhance the recognition and adherence between myoblasts, which allows alignment. It has been found that myoblast recognition and fusion is associated with the up-regulation of specific membrane proteins and with a change in phospholipids and ganglioside composition of the external leaflet of plasma membranes [68]. We speculate that TMZ, by acting on lipid β-oxidation, might reorganize the phospholipid composition of sarcolemma, this enhancing alignment of myoblasts [69].

Taken together our data show that the metabolic modulator TMZ promotes new myofiber formation. Metabolism modulation is emerging as a major driver of the stem cell regenerative capacity and designing new strategies such as TMZ able to direct metabolism optimization in SCs might have a high impact on aging and regeneration-impaired disorders, including cachexia, as well as having implications in regenerative medicine approaches. Moreover, this effect on myogenesis might contribute to the modulation of protein synthesis and degradation mechanisms, previously detected for TMZ [47, 48]. This would enhance this drug’s potential repositioning and therapeutical use in fighting skeletal muscle wasting in chronic pathologies, e.g. as a rehabilitation tool when the conditions of the patient do not allow exercise or in association with other treatment to facilitate functional recovery.

## MATERIALS AND METHODS

### C2C12 cell culture and treatments

Murine C2C12 skeletal myoblasts (American Type Culture Collection; ATCC, Manassas, USA) tested for contamination were grown at 37°C in a 5% CO_2_ in an air humidified chamber in high glucose DMEM (Dulbecco’s Modified Eagle’s Medium, Thermofisher, Monza, Italy) with Glutamax, supplemented with 20% FBS and 1% penicillin/streptomycin (growth medium; GM). As the cells approached confluency, the GM was replaced with a differentiation medium (DM; DMEM supplemented with 2% horse serum). All reagents for media preparation were from Thermo Scientific. The medium was changed every second day. The DM incubation was performed with or without TMZ (Servier, Rome, Italy; Alfa Aesar Thermofisher, Monza, Italy). After ascertaining the instability of the TMZ powder, we always used fresh TMZ, not older than one month after the opening of the powder package.

### Satellite cell isolation, culture and treatments

Excised muscles were transferred into a plastic Petri dish with cold PBS and visible adipose and connective tissues were removed with a scalpel. Muscles were cut into small pieces using scissors and the shredded muscle pieces were treated using the Skeletal Muscle Dissociation Kit (Miltenyi Biotec, Calderara di Reno-Bologna, Italy) according to the manufacturer’s instructions in order to isolate satellite cells. Purity of the suspension was checked by flow cytometry by labeling cells with anti-alfa7 integrin-PE conjugated antibody (Miltenyi Biotech) and ranged from 88% to 99% (Supplementary Figure 2). The purified cells were then centrifuged, resuspended in DMEM supplemented with 10% FBS, 20% HS and 3% CEE, and seeded in collagen precoated 35 mm dishes at 37°C under 5% CO_2_. After two days, medium was changed and cells were treated or not with TMZ.

### Fluorescence microscopy

Cells were treated as previously described [70] and stained with anti-MyHC 1:5 (MF20-Developmental Studies Hybridoma Bank at the University of Iowa), Myo D 1:100 (clone 5.8A, M3512, Dako, Agilent Technologies, Santa Clara, CA, USA) and Myogenin 1:100 (clone F5D, M3559, Dako) antibodies for 1 hour at RT. Cells were then washed in blocking buffer and incubated for 1 hour at 37°C with labeled anti-mouse 1:500 (Alexa Fluor 488; Molecular Probes, ThermoFisher) secondary antibody. Nuclei were stained with Hoechst 33342 (Sigma-Aldrich, Milan, Italy). The samples were mounted in FluoroMount mounting medium (Sigma-Aldrich). The images were acquired with an Olympus BX51/BX52 system microscope equipped with a Zeiss charge-coupled device (CCD) camera (Carl Zeiss, Milan, Italy).

### Cell orientation measurement

Images of stained cells were used to study directional analysis of myocytes/myotubes. A segment crossing the longest part of each cell was added and used to analyze the distribution of cell direction by the OrientationJ (Distribution) plug-in of Image J. OrientationJ computes the local orientation and the local coherency (anisotropy factor) of an image. The coherency index r can range from 0 (a perfect isotropy which is the existence of multiple cell directions) to 1 (in case of a perfect anisotropy which is the existence of a preferred-aligned-cell direction) [51].

### Quantitative real-time PCR

C2C12 were lysed with RLT from RNeasy Mini Kit (Qiagen, Hilden, Germany) and RNA extracted following manufacturer’s instructions. For RT-PCR, cDNA was synthesized with oligo-dT by adding 1μg of RNA with GoScript Reverse Transcription System (Promega, Madison, Wisconsin, USA). Comparative real-time PCR was performed with the SYBR-green master mix (Promega) using the Stratagene MX3000 (Thermo Fisher Scientific). Data were normalized to 18S and a calibrator was used as internal control. Resulting data were analysed as indicate in by the MX3PRO software (v4.10) and fold-change was determined by using the 2^-ΔΔCT^ method as as previously described [71]. All reactions were performed in triplicate. The following primers were used:

**Table:** 

18S	Fw 5’-CCCTGCCCTTTGTACACACC-3’ Rv 5’-CGATCCGAGGGCCTCACTA-3’
MyoD	Fw 5’-CCCCGGCGGCAGAATGGCTACG-3’ Rv 5’-GGTCTGGGTTCCCTGTTCTGTG-3’
Myogenin	Fw 5’-GGGCCCCTGGAAGAAAAG-3’ Rv 5’-AGGAGGCGCTGTGGGAGT-3’
MyHC	Fw 5′-CAAGTCATCGGTGTTTGTGG-3′ Rv 5′-TGTCGTACTTGGGAGGGTTC-3′
Desmin	Fw 5’-GAGGTTGTCAGCGAGGCTAC-3’ Rv 5’-GAAAAGTGGCTGGGTGTGAT-3’
Pax7	Fw 5’-CAAGCCCTGAGTCTCCTCAC -3’ Rv 5’-CATGGGTAGATGGCACACTG-3’
MCP-1	Fw 5’-CTTCTGGGCCTGCTGTTCA-3’ Rv 5’-CCAGCCTACTCATTGGGATCA-3’
Atrogin-1	Fw 5′-ATGCACACTGGTGCAGAGAG-3′ Rv 5′-CCTAAGGTCCCAGACATCCA-3′
MuRF-1	Fw 5’-GACAGTCGCATTTCAAAGCA-3’ Rv 5’-AACGACCTCCAGACATGGAC-3’
Myostatin	Fw 5’-CTGTAACCTTCCCAGGACCA-3’ Rv 5’-TCTTTTGGGTGCGATAATCC-3’

### Protein isolation and western blotting

Cells were washed twice in ice-cold PBS and lysed at 4°C in RIPA lysis buffer (50mM Tris/HCl, pH 8, 150mM NaCl, 0.5% sodium-deoxycholate, 1mM EDTA, 0.1% NP40, 0.1% SDS) supplemented with Phosphatase Inhibitor Cocktail 2 and 3 (Sigma-Aldrich) and Complete Protease Inhibitor (Roche, Monza, Italy). A clear supernatant was obtained by centrifugation of lysates at 13,000 g for 20 min at 4°C. Protein concentration in the supernatant was determined by Bradford protein assay (Bio-Rad). Aliquots of total lysates were then separated by SDS-PAGE using Miniprotean precast gels (BioRad) and proteins were transferred to nitrocellulose membranes (BioRad) or to polyvinylidene difluoride (PVDF) membranes (BioRad). Membranes were blocked 1h at RT with 5% non-fat milk in T-TBS (Tris-Buffered Saline with 0.05% Tween 20). Incubation with primary specific antibodies and horseradish peroxidase-conjugated secondary antibodies was performed in blocking solution for 1 hour at RT or over night at 4°C. We used antibodies against Laminin 1:1000 (L9393; Sigma-Aldrich), MyHC 1:20 (MF20; Developmental Studies Hybridoma Bank at the University of Iowa), slow MyHC 1:1000 (M8421; Sigma-Aldrich), LC3 1:1000 (MBL–Medical & Biological Laboratories, Nagoya, Japan); PARP 1:1000 (9542; Cell Signalling, Danvers, MA, USA), Caspase 3 1:1000 (05-654; Upstate Biotech, Lake Placid, NY, USA) [72], p62 1:1000 (pm045; MBL), Myo D 1:1000 (clone 5.8A, Dako, M3512), Myogenin 1:1000 (clone F5D, Dako, M3559), Pax7 1:1 (DSHB), pAMPK 1:1000 (2535; Cell Signaling) [71] and PGC1ɑ 1:1000 (AB3242; Millipore). The appropriate secondary horseradish peroxidase-conjugated antibodies from Jackson Immunoresearch were used in blocking solution (1:3000). Immunoreactive bands were visualised by SuperSignal West Pico Chemioluminescent substrate kit (Pierce). Equal loading of samples was confirmed by α-tubulin 1:1000 (T5168; Sigma-Aldrich) and bands quantified by densitometry using the ImageQuant TL software from GE Healthcare Life Sciences.

### BrdU incorporation assay

5-bromo-2’-deoxyuridine (BrdU) incorporation assay was performed by using a commercial kit (Cell Proliferation kit, Ge Healthcare). Cells were seeded in P35 dishes (45 10^3^ cells) in GM. After 48 hours, cells were treated or not for 24 h with TMZ. Alternatively, cells were seeded in P35 (90X10^3^ cells) in GM. After 48 h, medium was replaced by differentiation medium DM and cells treated or not for 24 hwith TMZ. During the last 4 h of treatments, BrdU labelling solution was added to cultures. After 30’ of fixation with fixative/denaturing solution (95% ethanol/0.1% acetic acid), cells were incubated with anti-BrdU monoclonal antibody for 1 h at RT. Cells were then washed with PBS and incubated with a FITC-labeled secondary antibody (Molecular Probes, ThermoFisher) for 1 h at RT. After three final washes with PBS, cells were incubated with DAPI (Vector Laboratories, Peterborough, UK). Images were collected with a Nikon Eclipse TE 2000U fluorescence microscope and analyzed with ImageJ software.

### Cell cycle analysis

Cell cycle was analyzed using propidium iodide (PI) (Sigma-Aldrich) staining. Briefly, after treatment with TMZ, cells were harvested using 0,05% Trypsin-EDTA, washed two times in PBS and fixed with 70% cold ethanol. Cells were incubated 30 min at -20°C, then ethanol was removed and cells were resuspended in a PBS containing 50 μg/ml PI for 30 min at 4°C in the dark. Cells were then washed, resuspended in PBS, acquired and analyzed on a FACScalibur flow cytometer (Becton Dickinson) using CellQuest software.

### Measurement of reactive oxygen species production

The reactive oxygen species (ROS)-sensitive probe DCF-DA (2’,7’-dichlorodihydrofluorescein-diacetate 30 μM) was added to the culture medium for 1 h. Cells were harvested using 0,05% Trypsin-EDTA, then washed with PBS and analyzed immediately by flow cytometry using the FITC channel on a FACScalibur flow cytometer (Becton Dickinson, Milan, Italy).

### Animals and experimental design

The animals were were obtained from Envigo S.R.L and housed on a 12-hour light/dark cycle with free access to a standard chow diet and water *ad libitum* during the whole experimental period, including the night before sacrifice. All procedures were approved by the ethics committee of Sapienza University of Rome-Unit of Histology and Medical Embryology and were performed in accordance with the current version of the Italian Law on the Protection of Animals. Experimental animals were cared for in compliance with the Italian Ministry of Health Guidelines (n° 86609 EEC, permit number 106/2007-B) and the Policy on Humane Care and Use of Laboratory Animals (NIH 1996).

### C26 tumor-bearing mice

Balb-c male mice were randomized according to their body weight on the day before the treatments and divided into four groups, namely controls and tumor bearers, treated or not with the trimetazidine (TMZ). The number of the animals was chosen by a sample size simulation which allows a 85% power to detect the difference found in a previous study [14]. In more detail, the first group (Ctrl; n=6) served as controls and included healthy mice inoculated with vehicle (saline); the second group (TMZ; n=6) included healthy mice receiving intraperitoneal (i.p.) injection of 5mg/kg TMZ once a day for 12 consecutive days; the third group (C26; n= 6) included tumor-bearing mice inoculated subcutaneously (s.c) dorsally with 5X10^5^ C26 carcinoma cells; the fourth group (C26-TMZ; n= 6) was inoculated with C26 cells and the same day started receiving TMZ i.p. injection of 5mg/kg TMZ once a day for 12 consecutive days. The day of sacrifice (12 days after tumor transplantation) the animals were anesthesized by isoflurane inhalation. Mice were then sacrificed by cervical dislocation and *gastrocnemius* (GSN) muscles were rapidly excised, weighed and frozen in liquid N_2_-cooled isopentane and finally stored at -80°C. GSN were homogenized by using a GentleMACS Dissociator (Miltenyi Biotec) and lysed in ice cold lysis buffer (10 mM Tris/HCl, pH 7.4, 5 mM EDTA, 5 mM EGTA, 1% Triton X-100, 130 mM NaCl, 0.1% SDS, 0.1% sodium deoxycholate).

### Cardiotoxin-induced muscle regeneration

For the cardiotoxin muscle-crush injury/regeneration evaluation, 3 months old male C57BL/6 mice were used. Mice were anesthetized with an intraperitoneal injection of physiologic saline containing Avertin (250 mg/Kg) and 10μM cardiotoxin (CTX) (Latoxan L81-02-isolated from Naja pallida nigricollis snake venom) was then intramuscularly administered into the right TA in a volume of 60 μl with a 27-gauge needle. The contralateral untreated TA muscle served as control. For TMZ-treatment, animals were injected with 5mg/kg body weight/die TMZ in PBS or PBS alone (daily, from -1 to +4 days with respect to muscle injury date T0). CTX-injured either PBS or TMZ-treated mice, were sacrificed at 5 and 15 days after cardiotoxin injection and TA muscles were rapidly excised and frozen in liquid N2 and finally stored at -80°C. Dissociation of muscles was performed with Qiagen TissueRuptor and RNA isolation was performed using TRIreagent (Sigma-Aldrich) following manufacturer’s instructions.

### Skeletal muscle immunofluorescence and morphometry

Serial muscle sections (9 μm) were obtained from the midbelly region of the TA muscles embedded in OCT (Bio-Optica, Milan, Italy) and snap frozen in liquid nitrogen-cooled isopentane (VWR International, Radnor, Pennsylvania, USA). A CM1900 cryostat (Leica, Wetzlar, Germany) at -20°C was used. Sections were fixed in 4% paraformaldehyde for 10 min and washed 3 times with PBS for 2 min. After permeabilization with 0.2% Triton X-100 (Sigma-Aldrich) and 1% BSA (Sigma-Aldrich) in PBS for 30 min, the samples were blocked for 1h in 10% horse serum/ PBS at RT and then incubated for 1 h with anti-laminin (L9393 Sigma-Aldrich) antibody diluted in PBS (1:1000). The Alexa Fluor 488 anti rabbit IgG (A11008) from Thermo Fisher was used as secondary antibody. Nuclei were visualized with the DNA dye 40,6-diamidino-2-phenylindole (DAPI) (Thermo Fisher) and the samples were mounted in SlowFade Gold mounting media (Thermo Fisher). The images were acquired with a Leica TCS SP5 confocal microscope. In the stained muscle sections, automated CSA determination along the laminin-stained border of each fiber was evaluated by using Image J software (http://rsb.info.nih.gov/ij/) (NIH) [73]. To prevent errors in fiber border recognition (i.e. either the fibers might not be recognized or several fibers/non-fiber regions might be interpreted as a single fiber), a manual correction of myofiber border misinterpretation was performed.

### Statistics

All experiments were performed at least four times, unless otherwise indicated. Data are presented as mean ± standard error of the mean (s.e.m.). Statistical differences between groups were verified by Student’s *t*-test (2-tailed). ^*^P < 0.05 was considered significant.

## SUPPLEMENTARY MATERIALS FIGURES



## Abbreviations

AMP: adenosine monophosphate
AMPK: AMP-activated protein kinase
ATP: adenosine triphosphate
BrdU: 5-bromo-2’-deoxyuridine
BSA: bovine serum albumin
CEE: chicken embryo extract
CPT1: carnitine palmitoyl transferase 1
CR: caloric restriction
CSA: cross sectional area
CTX: cardiotoxin
DCF-DA: 2’,7’-dichlorodihydrofluorescein-diacetate
DM: differentiation medium
DMEM: Dulbecco’s Modified Eagle’s Medium
FAP: fibro-adipogenic progenitors
GM: growth medium
HS: horse serum
LC3: Microtubule-associated proteins 1A/1B light chain 3B
MCP1: Monocyte Chemoattractant Protein-1
Myf-5: myogenic factor 5
MyHC: myosin heavy chain
MyoD: myogenic differentiation
NAD: nicotinamide adenine dinucleotide
PARP: poly ADP ribose polymerase
PBS: phosphate buffered saline
PFA: paraformaldehyde
PGC1α: peroxisome proliferator-activated receptor gamma coactivator 1-alpha
PI: propidium iodide
ROS: reactive oxygen species
SCs: satellite cells
SIRT1: sirtuin (silent mating type information regulation 2 homolog) 1
SMF: static magnetic field
TA: tibialis anterioris
TMZ: trimetazidine
TNN: troponin
