# Supplementary material for: Modulating the metabolism by trimetazidine enhances myoblast differentiation and promotes myogenesis in cachectic tumor-bearing c26 mice
Source: Oncotarget. 2017 Dec 8;8(69):113938–56. doi: 10.18632/oncotarget.23044 (PMC5768376; doi:10.18632/oncotarget.23044)
Supplement: Supplementary file 1 [file oncotarget-08-113938-s001.pdf]

Modulating the metabolism by trimetazidine enhances myoblast differentiation and promotes myogenesis in cachectic tumor-bearing c26 mice

SUPPLEMENTARY MATERIALS

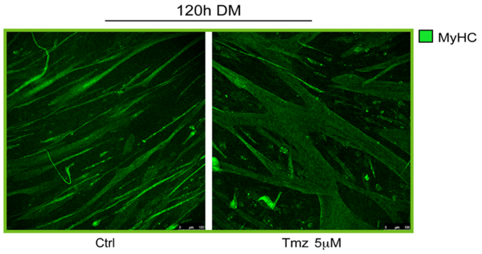

Supplementary Figure 1: TMZ robustly enhances C2C12 myotube size Representative pictures of C2C12 myoblasts differentiating for 120 hours in differentiating medium (DM) in the absence (Ctrl) or presence (TMZ) of 5mM TMZ and then stained with anti-MyHC antibody (green).

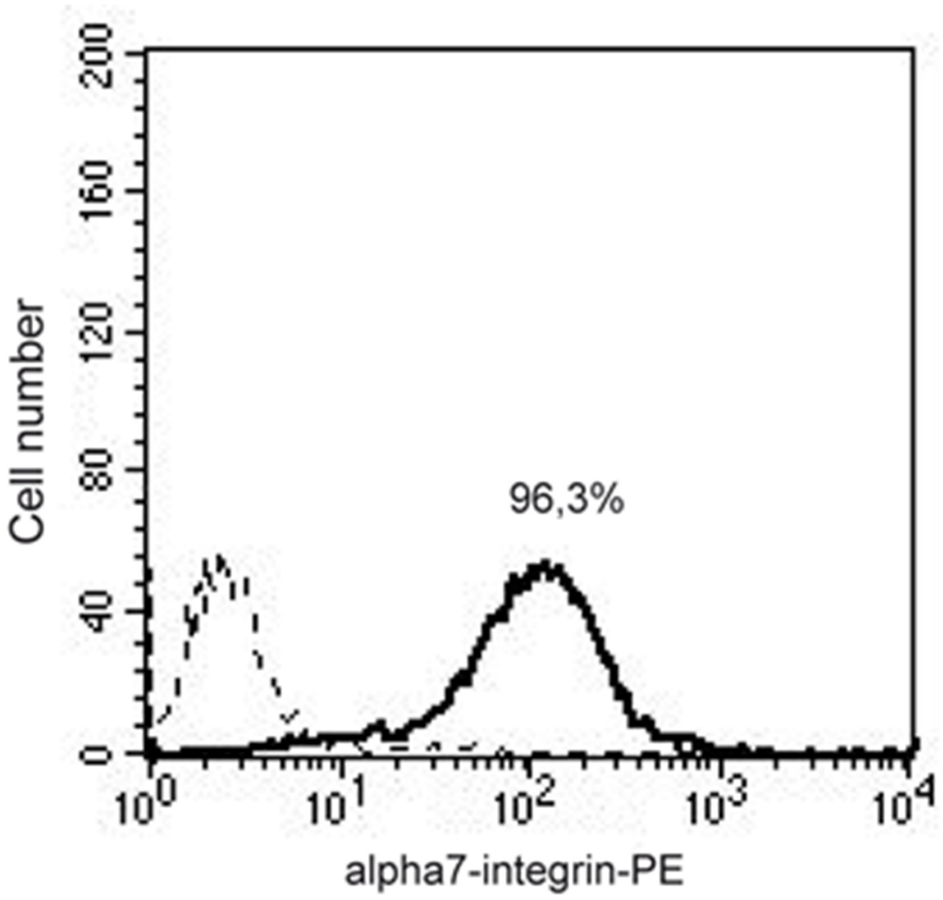

Supplementary Figure 2: Purity of isolated satellite cells. Freshly isolated satellite cells were stained with an anti-alpha 7 integrin antibody, then acquired and analyzed by flow cytometry. Histogram plots show the expression of alpha 7 integrin (solid line) on satellite cells. Dotted line, isotype control. Numbers in plots indicate the percentage of alpha 7 integrin expressing cells. The plot is representative of 3 different experiments.

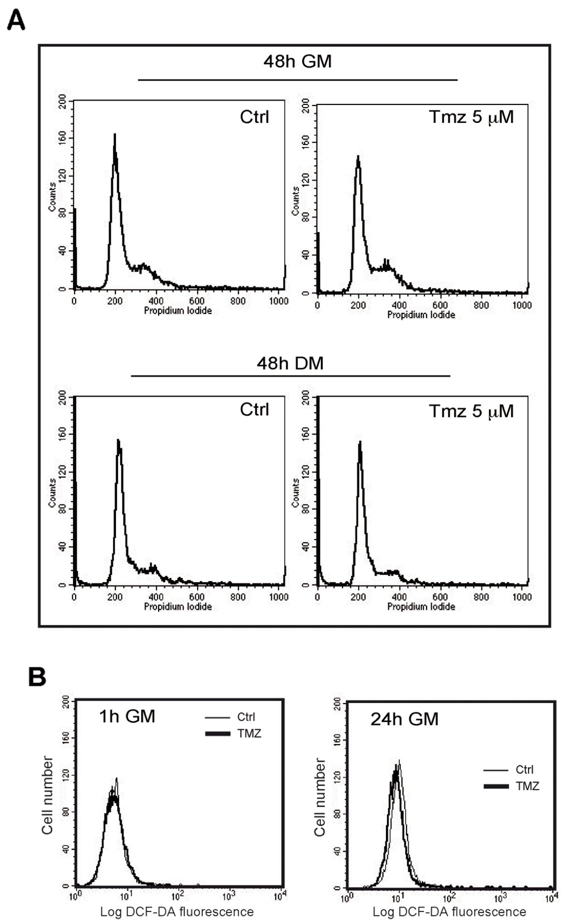

Supplementary Figure 3:TMZ does not affect C2C12 proliferation rate or ROS production. **(A)** Cell cycle analysis by propidium iodide (PI) staining and FACS analysis in GM- and DM-incubated C2C12 cells in presence or absence of TMZ 5 μM for 48 hours. No differences were observed in the percentage of resting cells (G0/G1) nor in that of proliferating cells (S/G2). **(B)** 1 hour or 24 hours DM differentiating myoblasts untreated (Ctrl), or treated with TMZ (TMZ) for 1 hour or 24 hours underwent ROS measurement by FACS analysis. One hour before the analysis, cells were incubated with the ROS-sensitive probe DCF.

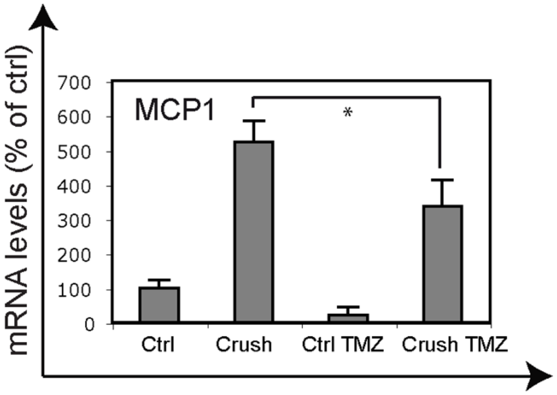

Supplementary Figure 4: cytokine MCP1 tend to decrease following TMZ treatment. The mRNA levels of MCP1 were evaluated by quantitative real time PCR and were normalised to 18S used as internal control in TA muscles of untreated control mice (Ctrl), cardiotoxin (CTX)-injured mice (Crush), TMZ-treated control mice (Ctrl TMZ), and TMZ-treated CTX-injured mice (Crush TMZ) 5 days post-injury. Data display the percentage of mRNAs relative to control, which was arbitrarily set as 100. Data shown are the mean ± SEM from three experiments each performed in triplicate. $^{*}p \leq 0.05$ by Student's *t*-test.

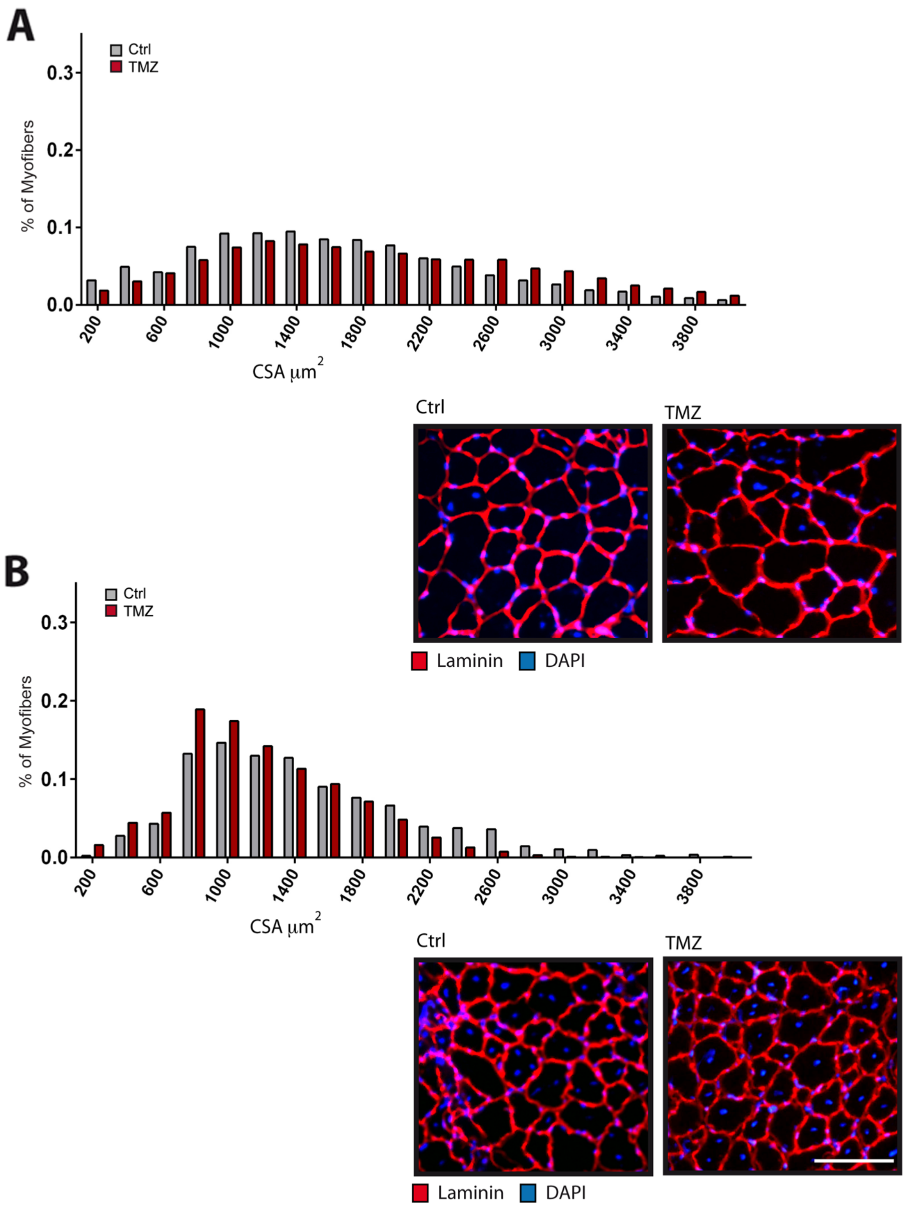

Supplementary Figure 5: Frequency histograms on total myofibers. **(A)** Muscle sections of the CTX-injected muscles were analyzed 15 days post-injury. Representative images of whole Laminin/DAPI stained cross-sections from TA muscles damaged with CTX untreated (Ctrl) or treated (TMZ) with TMZ are shown. Five untreated mice and and five TMZ-treated mice and at least 5000 myofibers from 5 untreated mice and at least 5000 myofibers from 5 TMZ-treated mice have been evaluated for the determination of cross-sectional area (CSA). Frequency histograms show the distribution of CSA measured for total myofibers. Scale bar: 100 μm. **(B)** Same as (A) but at 10 days post-injury.
